# Catalyzing the publication of international research in child and adolescent mental health

**DOI:** 10.1186/1753-2000-7-23

**Published:** 2013-07-31

**Authors:** Christian Kieling, Andrés Martin

**Affiliations:** 1 Department of Psychiatry, Hospital de Clínicas de Porto Alegre, Universidade Federal do Rio Grande do Sul, Rua Ramiro Barcelos, 2350 - 400N, Porto Alegre, RS, 90350-009, Brazil; 2 Child Study Center, Yale School of Medicine, 230 South Frontage Road, New Haven, CT, 06520, USA

## 

*Child and Adolescent Psychiatry and Mental Health (CAPMH)* has recently become the official journal of the International Association for Child and Adolescent Psychiatry and Allied Professions (IACAPAP). This joint venture can have profound implications for the flourishing of scientific research on child and adolescent mental health where it is most needed – in low- and middle-income countries. Although nine out of ten individuals under the age of 18 years live in these nations, only 10% of the articles published on the mental health of children and adolescents come from these regions of the world [1].

The relatively new area of child and adolescent mental health has demonstrated to be of increased interest to the scientific community, as evidenced by the rising number of journal articles over the last decade, jumping from fewer than five thousand to more than ten thousand indexed items per year in the period from 2002 to 2011 [2]. Research conducted in areas such as epidemiology, clinical presentation and interventions on mental disorders early in life is indispensable to strengthen the scientific bases of child and adolescent mental health clinical practice. In this sense, knowledge disseminated by scientific journals lays the foundations for textbooks such as the *IACAPAP Textbook of Child and Adolescent Mental Health*[3].

In comparison to its more traditional journals in the field of child and adolescent mental health, *CAPMH* is a younger sibling that has already demonstrated a sound scientometric performance. This phenomenon can be seen in the SCImago Journal Rank (SJR), a measure of scientific influence that takes into account not only the number of citations received by a journal, but also the prestige of the journals that granted such citations [4]. The most recent SJR for *CAMPH* was 0.788, representing a fivefold increase over the last four years. In the latest assessment, leading journals in the field such as the *Journal of the American Academy of Child and Adolescent Psychiatry*, the *Journal of Child Psychology and Psychiatry*, and the *European Child and Adolescent Psychiatry* exhibited SJRs of 2.535, 2.344, and 1.008, respectively.

Beyond citation counts, there are many other considerations that are important in assessing the relevance of scholarly publishing. The peer review process has always been conceptualized as essential to ensure the trustworthiness of scientific research. It is by submitting someone’s work to the scrutiny of peers that editors make most decisions on whether a manuscript should be published or not. In fact, one could even argue that the peer review process is the *rate-limiting step* in the generation of high-quality research in any field of science – in chemical kinetics, the velocity of a reaction with several steps is often determined by this crucial stage, which limits the speed of the entire mechanism.

Peer review has a primary function of “improving the process and the coherence of scientific knowledge and its utility” [5]. In addition to the geographical imbalance in the authorship of papers focusing on the mental health of children and adolescents worldwide, the limited representation of reviewers from less resourced nations also imposes barriers that ultimately result in a reduced representation of the research output in a global perspective. Having an editorial board with a diverse international representation (including 43 members from 14 countries), *CAPMH* widens the comprehensiveness of the scientific community involved in publishing processes related to child and adolescent mental health.

Traditional print journals have space constraints, limiting the number of articles that can be published. This has lead not only to the advent of open access publishers, such as BioMed Central, and online repository-type journals such as the PLoS ONE (part of the Public Library of Science and the biggest journal in the world, with 23,468 papers published in 2012), but also to the flourishing of “open access” practices. These initiatives intend to remove barriers (e.g., subscription costs) to the dissemination of knowledge, transferring to the author (or his/her funding agency) the expenses involved in the publication process. Although such a model, in which authors are charged to get their work published, is potentially vulnerable to misuse, robust peer review once again becomes a key ingredient to ensure the quality of published research. A recent report by the UK Parliament concluded that experiences such as the PLoS ONE “will accelerate the pace of research communication and ensure that all work that is scientifically sound is published” [5].

Interestingly, in recent years journals from traditional publishing houses are increasingly adopting open access strategies. In addition to offering authors the option to pay in order to have their manuscript available free of charge, many journals have recently adhered to other more broad ventures. One example is the World Health Organization HINARI Program, which provides access to 150 publishers, including more than 10,000 journals and 18,500 books to local, not-for-profit institutions in less resourced countries. Further information and a list of countries with free (Group A) or reduced cost (Group B) access can be found at the HINARI Access to Research in Health Programme [6]. The US National Institute of Health (NIH) PubMed Central digital archive is another initiative working under the free access principle, including entire journal collections and also author manuscripts of journal articles that were supported by NIH funding [7].

As an online-open access journal, *CAPMH* ensures visibility to an extensive readership worldwide; as the official journal of IACAPAP, it is well positioned to catalyze global collaborative efforts. One early example of this potential synergy is the series of articles on the theme of identity published in this issue of *CAPMH*[8-13]. These articles were the direct outgrowth of fruitful discussions held in the Paris 2012 IACAPAP Congress.

*CAPMH* provides an international platform for fast and ample scientific communication on youth mental health across distinct cultural backgrounds. *CAPMH* welcomes research from underrepresented areas and, to ensure the publication of such manuscripts, the journal’s publisher, BioMed Central, has an open access waiver fund, whereby they provide an automatic waiver of the article processing charge for authors based in low- and lower-middle-income countries [14]. Additionally, BioMed Central has a Membership Program and authors whose institution has joined this program have their article processing charge covered in full or in part by the membership [15]. *CAPMH* is hence in a privileged position to catalyze the research output from multiple parts of the globe, including those where most children and adolescents currently live.

## References

[B1] Kieling C Baker-Henningham H Belfer M Conti G Ertem I Omigbodun O Rohde LA Srinath S Ulkuer N Rahman A Child and adolescent mental health worldwide: evidence for action Lancet 2011 378 1515 1525 10.1016/S0140-6736(11)60827-1 22008427

[B2] Kieling C Rohde LA Child and adolescent mental health research across the globe J Am Acad Child Adolesc Psychiatry 2012 51 945 947 10.1016/j.jaac.2012.07.002 22917207

[B3] Rey JM IACAPAP e-Textbook of Child and Adolescent Mental Health 2012 Geneva: International Association for Child and Adolescent Psychiatry and Allied Professions http://iacapap.org/iacapap-textbook-of-child-and-adolescent-mental-health

[B4] Butler D Free journal-ranking tool enters citation market Nature 2008 451 6 10.1038/451006a 18172465

[B5] House of Commons, Science and Technology Committee Peer review in scientific publications Eighth Report of Session 2010–12. Volume I: Report, together with formal minutes, oral and written evidencehttp://www.publications.parliament.uk/pa/cm201012/cmselect/cmsctech/856/856.pdf20666769

[B6] HINARI access to research in health programme http://www.who.int/hinari/en/

[B7] PubMed Central http://www.ncbi.nlm.nih.gov/pmc

[B8] Schmeck K Fegert JM Schlüter-Müller S On identity Child Adolesc Psychiatry Ment Health 2013 7 24 10.1186/1753-2000-7-24 23899368 PMC3766016

[B9] Kassin M de Castro F Arango I Goth K Psychometric properties of a culture-adapted Spanish version of AIDA (Assessment of Identity Development in Adolescence) in Mexico Child Adolesc Psychiatry Ment Health 2013 7 25 10.1186/1753-2000-7-25 23899385 PMC3765088

[B10] Jung E Pick O Schlüter-Müller S Schmeck K Goth K Identity development in adolescents with mental problems Child Adolesc Psychiatry Ment Health 2013 7 26 10.1186/1753-2000-7-26 23899433 PMC3751041

[B11] Schmeck K Schlüter-Müller S Foelsch PA Doering S The role of identity in the DSM5 classification of personality disorders Child Adolesc Psychiatry Ment Health 2013 7 27 10.1186/1753-2000-7-27 23902698 PMC3848950

[B12] Northoff G Brain and self - A neurophilosophical account Child Adolesc Psychiatry Ment Health 2013 7 28 10.1186/1753-2000-7-28 23902725 PMC3734106

[B13] Sollberger D On identity: from a philosophical point of view Child Child Adolesc Psychiatry Ment Health 2013 7 29 10.1186/1753-2000-7-29 PMC3751052 23902741

[B14] Open access waiver fund http://www.biomedcentral.com/authors/oawaiverfund/

[B15] About membership http://www.biomedcentral.com/libraries/membership

